# Clinical and Biological Variables Influencing Outcome in Patients with Advanced Non-Small Cell Lung Cancer (NSCLC) Treated with Anti-PD-1/PD-L1 Antibodies: A Prospective Multicentre Study

**DOI:** 10.3390/jpm12050679

**Published:** 2022-04-24

**Authors:** Erica Quaquarini, Federico Sottotetti, Francesco Agustoni, Emma Pozzi, Alberto Malovini, Cristina Maria Teragni, Raffaella Palumbo, Giuseppe Saltalamacchia, Barbara Tagliaferri, Emanuela Balletti, Pietro Rinaldi, Costanza Canino, Paolo Pedrazzoli, Antonio Bernardo

**Affiliations:** 1 Medical Oncology Unit, ICS Maugeri-IRCCS SpA SB, 27100 Pavia, Italy; federico.sottotetti@icsmaugeri.it (F.S.); cristina.teragni@icsmaugeri.it (C.M.T.); raffaella.palumbo@icsmaugeri.it (R.P.); giuseppe.saltalamacchia@icsmaugeri.it (G.S.); barbara.tagliaferri@icsmaugeri.it (B.T.); emanuela.balletti@icsmaugeri.it (E.B.); antonio.bernardo@icsmaugeri.it (A.B.); 2 Medical Oncology Unit, IRCCS San Matteo Hospital Foundation, 27100 Pavia, Italy; f.agustoni@smatteo.pv.it (F.A.); costanza.canino01@universitadipavia.it (C.C.); p.pedrazzoli@smatteo.pv.it (P.P.); 3 Oncology Unit, Ospedale Civile, 27058 Voghera, Italy; emma.pozzi01@gmail.com; 4 Laboratory of Informatics and System Engineering for Clinical Research, ICS Maugeri-IRCCS SpA SB, Via Maugeri 10, 27100 Pavia, Italy; alberto.malovini@icsmaugeri.it; 5 Department of Internal Medicine and Therapeutics, University of Pavia, 27100 Pavia, Italy; 6 Unit of Thoracic Surgery, IRCCS San Matteo Hospital Foundation, 27100 Pavia, Italy; p.rinaldi@smatteo.pv.it

**Keywords:** advance stage, anemia, immune checkpoint inhibitors, non-small cell lung cancer

## Abstract

Introduction: Immune checkpoint inhibitors (ICIs) have become the standard of treatment for patients with non-small cell lung cancer (NSCLC). However, there are still many uncertainties regarding the selection of the patient who could benefit more from this treatment. This study aims to evaluate the prognostic and predictive role of clinical and biological variables in unselected patients with advanced NSCLC candidates to receive ICIs. Methods: This is an observational and prospective study. The primary objective is the evaluation of the relationship between clinical and biological variables and the response to ICIs. Secondary objectives included: safety; assessment of the relationship between clinical and biological parameters/concomitant treatments and progression-free survival at 6 months and overall survival at 6 and 12 months. Nomograms to predict these outcomes have been generated. Results: A total of 166 patients were included. An association with response was found in the presence of the high immunohistochemical PD-L1 expression, squamous cell histotype, and early line of treatment, whereas a higher probability of progression was seen in the presence of anemia, high LDH values and neutrophil/lymphocyte ratio (NLR), pleural involvement, and thrombosis before treatment. The nomogram showed that anemia, PD-L1 expression, NLR, and LDH represented the most informative predictor as regards the three parameters of interest. Conclusions: In the era of personalized medicine, the results are useful for stratifying the patients and tailoring the treatments, considering both the histological findings and the clinical features of the patients.

## 1. Introduction

Lung cancer (LC) is one of the most common tumors in the world [1]. It is the first cancer in the male sex and the third in women. Prognosis is poor and depends on the clinical stage at diagnosis; in the metastatic setting, the 5- and 10-year survival is 16% and 12%, respectively. Smoking is the main risk factor, and it represents the cause of 85–90% of LC diagnosed. Non-small cell lung cancer (NSCLC) is the most common subtype [2]. The identification of alterations in components of signal transduction pathways, leading to sustained tumor growth and survival, has changed the possibility of treatment of the oncogene-addicted NSCLC [3]. However, patients resulting in the wild type (WT) for driver molecular alterations cannot be treated with targeted therapies. LC was traditionally considered a nonimmunogenic tumor, but advancements in tumor immunology and recent immunotherapeutic success have changed this view [4]. Indeed, chemotherapy and immune checkpoints inhibitors (ICIs) currently represent the main treatment for patients with WT NSCLC. In this evolving scenario, in Italy, platinum-based chemotherapy plus ICI is the standard first-line in metastatic NSCLC and programmed-death ligand 1 (PD-L1) expression < 50%, while in the presence of PD-L1 ≥50%, the indication is usually to immunotherapy with pembrolizumab, an IgG4 antibody that targets the programmed cell death protein-1 (PD-1) [5]. Immunotherapy is also approved as a subsequent treatment line as, independently of PD-L1 status, patients progressing to first-line chemotherapy can be candidates for nivolumab, an IgG4 monoclonal antibody that blocks PD-1, or atezolizumab, a humanized IgG1 monoclonal antibody directed against the PD-L1, while patients with PD-L1 expression ≥1% can receive pembrolizumab.

The randomized phase II/III studies that led to the approval of these drugs for use in clinical practice have described improved objective responses, time to progression, and overall survival (OS) compared to standard chemotherapy in a limited number of selected patients [6]. The fast-growing number of ICIs and the uncertainties regarding the selection of the patient who could benefit more from these treatments strengthen the need for predictive biomarkers. In a context in which LC prognosis has shown a slow but constant improvement, the importance of those “clinical” variables linked to the patient and his non-oncological anamnesis is increasing since they contribute to predicting the benefit of the active cancer treatments as well as their potential toxic effects [7]. In the current epidemiological context characterized by an aging population and the consequent increase in the prevalence of chronic degenerative diseases, the phenomenon of “multimorbidity” is increasingly common in clinical practice [8]. A condition of multimorbidity significantly modifies both the clinical expression of disease conditions and response to prescribed treatments, and it is associated with a state of clinical complexity that can be at least partially assessed by currently adopted evaluation scales. This complexity, which is mostly represented in the unselected patients of the daily clinical practice, may determine alterations in the body’s homeostatic systems and drug interactions [9,10].

The present study aims to evaluate the prognostic and predictive role of biological and clinical variables in unselected patients affected by advanced NSCLC and treated with ICI monotherapy in order to elaborate a useful tool to assist in the decision-making process.

## 2. Patients and Methods

### 2.1. Characteristics of the Study

This is an open-label, prospective, observational multicentre study conducted between June 2016 and September 2020 in two oncology centers, Istituti Clinici Scientifici Maugeri (ICSM) and Policlinico San Matteo (PSM), located in Pavia city, Northern Italy. The study was conducted in accordance with the Declaration of Helsinki and approved by the Ethical Committee of the coordinating Institution (ICS Maugeri (ICSM) IRCCS Pavia Ethic Committee) and adopted by the satellite center (PSM). All patients provided written informed consent for the analysis and anonymized publication of clinical data.

### 2.2. Patients

We considered eligible patients with a cytological or histological diagnosis of locally advanced/metastatic NSCLC without driver-mutations (EGFR, ALK, ROS1) who were candidates to receive an ICI treatment as a single agent according to AIFA guidelines and clinician’s indication. Additional inclusion criteria were a measurable disease according to Response Evaluation Criteria In Solid Tumors (RECIST) criteria, version 1.1, and a minimum follow-up of 3 months. Patients could receive ICI in each treatment line; previous chemotherapy for metastatic disease was allowed. Data collection started from the administration of the first dose of immunotherapy and included: patients’ performance status (PS) evaluated using the Eastern Cooperative Oncology Group (ECOG) scale and age at the study entry; disease characteristics, sites and number of metastases, tumor histology, and immunohistochemical (IHC) expression of PD-L1 on cancer tissue; previous therapies received in adjuvant and metastatic setting; comorbidity status, evaluated by the Age-adjusted Charlson Comorbidity Index (ACCI) scale; LDH (mU/mL), neutrophil/lymphocyte ratio (NLR) and hemoglobin values before the study entry; smoking habits and line of treatment with ICIs; concomitant treatments, in particular oral or intravenous iron supplements, blood transfusions, antiplatelet/anticoagulants, erythropoietin, proton pump inhibitors (PPIs), steroids, and prior (within 15 days) or concomitant use of antibiotics. Anemia was defined on the basis of the regeneration pattern (regenerative versus hyporegenerative) and on the basis of the iron status. Functional or absolute iron deficiency anemia was defined if transferrin saturation (TSAT) < 20% and serum ferritin ≥ 30 or < 30 ng/mL, respectively. The status of vitamin B12 and folic acid has not been routinely evaluated as it is difficult to quantify and correctly interpret the levels of these analytes in patients with high cell turnover, and other metabolic products linked to vitamin B12 metabolism are not routinely dosed in clinical practice.

### 2.3. Immunotherapy Protocols

Nivolumab was administered at a dose of 240 mg once every 2 weeks as a 30 min infusion; pembrolizumab was administered at a dose of 200 mg once every 3 weeks as a 30 min infusion; atezolizumab was administered at a dose of 1200 mg once every 3 weeks as a 30 min infusion. Treatment was administered until documented disease progression (PD) or clinical PD, unacceptable toxicity, or patient refusal and was given in an outpatient setting, according to the officially approved national guidelines. The tumor assessment was performed with computed tomography (CT) scan approximately every four months unless there were clinical signs of PD, according to clinical practice and physician’s indication. Treatment efficacy was evaluated by immune Response Evaluation Criteria In Solid Tumors (RECIST version 1.1) [11]. Adverse events (AEs) were recorded and graded according to the National Cancer Institute Common Terminology Criteria for Adverse Events (NCI-CTCAE) (version 5.0) [12]. A complete blood count and organ function test was performed before each cycle. Dose delays were planned to correspond with the type and grade of observed toxicity, according to the summary of product characteristics. Concomitant medications such as antiemetic drugs and bisphosphonates were allowed; steroids were allowed at a maximum daily dose of 10 mg of prednisone or equivalent molecule.

### 2.4. Study Objectives

The primary objective of the study is to assess the association between the clinical-biological variables considered and the response to ICI.

Secondary objectives include: the evaluation of the safety and tolerability of the treatment; the association between concomitant medications and response to ICI; the association between the clinical and biological variables considered and time to progression and death. Multivariate models and visual methods have been generated to predict response, progression at 6 months, and death at 6 and 12 months from immunotherapy start.

### 2.5. Study Outcomes

The assessment of comorbidities was performed using the ACCI scale. The score was defined for all patients at baseline, before the start of the ICI therapy. ACCI is composed of 19 different categories with a variable score between 1 and 6 and a total maximum score of 33.

The overall response was defined as the best-confirmed response detected in each patient from the start to the end of ICI treatment. The following study endpoints have been defined: type of response (complete response (CR), partial response (PR), stable disease (SD), and PD); progression-free survival (PFS), defined as the time in months from the start of immunotherapy to the earliest date of tumor relapse or death for any cause; overall survival (OS), as the time in months from the beginning of immunotherapy to death for any cause or to the last date of follow-up. We defined a “responder” to ICI as a patient that obtained CR, PR, or SD during treatment.

### 2.6. Statistical and Machine Learning Methods

Statistical and machine learning analyses have been performed by the R statistical software tool version 4.0.5 (www.r-project.org accessed on 15 June 2021).

#### 2.6.1. General Statistical Methods

A preliminary phase of data quality control allowed checking for the presence of potential errors during data collection. Numeric variables were discretized according to clinically relevant thresholds, and their distribution was described by median (interquartile range, IQR), while categorical variables were distribution by absolute and relative frequencies (%). The Pearson chi-square test with simulations (*n* = 10,000 replicates) was used to compare categorical variables distribution between centers. Logistic regression was used to test for association between explanatory variables and binary response variables. The Cox proportional hazard method was used to assess the risk of events, while the log-rank test was used to compare survival profiles among subgroups. The center ID (ICSM/PSM) was included in the logistic regression and Cox proportional hazard regression models to adjust for confounders when analyzing combined datasets.

#### 2.6.2. Multivariate Models for Response Prediction

Multinomial group LASSO regression (*msgl* package) was used to identify the most informative subset of variables jointly informative with respect to the best response type. To this aim, different combinations of multi-level categorical variables and dichotomized categorical variables (Supplementary Tables S1 and S2) were tested within a 5-fold cross-validation (CV) schema using the ICSM dataset. More in detail, the ICSM dataset was split randomly into 5 folds with the best response stratification in order to preserve the same proportion of class values across folds. Different models (Supplementary Table S2) were trained after lambda value tuning (the lambda value guaranteeing the lowest binomial deviance by a further internal CV was chosen) on each ICSM CV training set and tested on the corresponding ICSM CV validation set not used for models fitting. The most informative model was then identified as the one reaching the highest mean area under the receiver operating characteristics curve (AUROC) over the 5 validation sets weighed by the sample size of each validation set. The most informative model was then trained on the whole ICSM dataset and tested on the PSM dataset, used as an independent test set. The discriminative performances on the test set were quantified in terms of global AUROC (*multiclass.roc*, *pROC* package) and pairwise AUROC (*roc* function, *pROC* package), sensitivity, specificity, positive predictive value (PPV) and negative predictive value (NPV) (*epi.tests* function, *epiR* package), and 95% confidence intervals (95% CI) comparing one class value against the other two in turn.

#### 2.6.3. Multivariate Models for PFS and OS Prediction

Cox regression with grouped LASSO penalties (*grpreg* package) was used to identify the most informative subset of variables jointly informative with respect to the progression and death survival outcomes (Supplementary Tables S1 and S2). Selected variables were then used to fit two multivariate Cox regression models (one by outcome). The same combinations of predictors used for best response type prediction were evaluated by a 5-fold CV schema within the ICSM dataset. The most informative model was then identified as the one reaching the highest weighted mean survival AUROC in predicting 6- and 12-month PFS/OS over the validation sets. The most informative model was then trained on the whole ICSM dataset and tested on the PSM dataset, used as an independent test set. Discriminative performances were quantified in terms of 6- and 12-month survival AUROC (*Score* function, *riskRegression* package). C statistic was estimated by the *BeggC* function implemented in the *survAUC* package. The *nomogram* function implemented in the *cph* package was used for nomograms generation and partial and total points computations. Survival trees (*rpart* function, *rpart* package) were used to identify cut-off values in terms of total points generated by the nomogram able to distinguish binary risk groups.

## 3. Results

### 3.1. Patient Characteristics

Over the study period, 166 patients were enrolled and treated (ICSM dataset: 95 patients and PSM dataset: 71 patients). The clinical and demographic characteristics of the patients are reported in Table 1.

The median age of the analyzed patients was 68.5 years (IQR = 12 years, min = 33 years, max = 84 years). The majority of patients (67.47%) were ≥ 65 years old, 77.71% were males, and 92.77% were active or former smokers. About half of the patients (45.18%) had an ECOG PS score of 0, while 43.37% had an ECOG PS score of 1 and 11.45% of 2 or 3. About 25% of patients had squamous NSCLC (SCC), while 75% had a non-squamous carcinoma, histotype adenocarcinoma (ADC). At the start of the immunotherapy treatment, most patients (76.51%) had a stage IV disease.

### 3.2. Primary Objective: Association between Clinical-Biological Variables and Response to ICI

A total number of 63 patients (37.95%) were characterized by PD as the best response, 44 (26.51%) PR, and 59 (35.54%) SD. No patients obtained CR as the best response. Results from logistic regression fitted on ICSM and PSM combined datasets adjusting by center allowed identifying variables statistically associated with the response to treatment outcome (Table 2).

In detail, patients with SCC histotype had a higher probability of having a response to ICI treatment as well as patients receiving ICI as the second line of treatment. Compared to patients with IHC PDL1 < 1%, patients with IHC PDL1 ranging 1–24%, 25–49%, and ≥ 50% shared significantly higher probability to obtain SD or PR (OR < 0.3, *p* < 0.05). Patients with anemia had a significantly higher probability of PD (OR = 24.16, *p* < 0.0001). Altered NLR had a prognostic effect since patients with NLR ≥ 5 were significantly more likely to have PD as the best response (OR = 11.21, *p* < 0.0001) compared to patients with NLR < 5. Similarly, patients with LDH ≥ 325 were more likely to be characterized by PD (OR = 29.01, *p* < 0.0001) compared to patients with LDH < 325. Finally, diagnosis of thrombosis before therapy start and presence of pleural metastases defined a higher risk of PD (OR = 3.46, *p* = 0.0043; OR = 6.95, *p* = 0.0040).

Results by center are reported in Supplementary Table S3.

### 3.3. Secondary Objectives

The safety and tolerability of ICI were the secondary objectives of the study. A median of 15 doses of immunotherapy was administered (min-max: 2–82). Among the 166 patients analyzed, 53 (31.93%) developed at least one side effect. There were 81 treatment-related AEs of any grade, 17 of which were G3/4 (21%). Fatigue was the most frequent AE (G1–2 = 31%, G3 = 4%), followed by gastrointestinal (G1–2 = 17%, G3 = 1%) and endocrine toxicity (G1–2 = 8%, G3 = 3%). Other rarer AEs were: skin toxicity (G1–2 = 9%, G3 = 1%), arthralgia (G1–2 = 3%, G3 = 3%), lung toxicity (G1–2 = 6%, G3 = 7%, G4 = 1%), myopathy (G1–2 = 1%), ocular (G1–2 = 1%) and hematologic (G1–2 = 3%, G3 = 1%) AEs. The mean time to onset of AEs was 9.3 weeks. Due to G3/4 lung toxicity, four patients needed treatment discontinuation. There was one treatment-related death. Results from logistic regression showed that the development of toxicities during ICI did not influence the probability of response. Regarding the association between concomitant medications and type response to ICI, patients treated with antiplatelet or anticoagulants drugs had a higher chance of PD (OR = 2.06, *p* = 0.0289). No association between the type of response to ICI and the prior or concomitant treatments with antibiotics and concomitant exposure to corticosteroids, PPIs, erythropoietin, oral or intravenous iron supplements as well as blood transfusions was found (*p* > 0.05, Table 2).

#### 3.3.1. Response Type Prediction

Multivariate multinomial grouped LASSO logistic regression was used to identify variables jointly informative with respect to the response type (PD, PR, or SD) using data from 95 patients from the ICSM dataset (PD = 43, 45.26%, PR = 30, 31.58%, SD = 22, 23.16%) with complete data for the starting set of variables reported in Supplementary Table S1. The features selection algorithm identified line of treatment, histotype, IHC PDL1 (binarized < 25% versus ≥ 25%), ECOG PS, anemia, thrombosis before therapy, NLR, LDH, metastases sites, and ACCI as the most informative subset of predictors of the outcome. The most informative model has been then trained on ICSM data and tested on data from 64 patients defining the PSM dataset (PD = 20, 31.25%, PR = 13, 20.31%, SD = 31, 48.44%) with complete information for the set of variables included in the final multivariate model: the overall AUROC in discriminating among the three classes was 0.86. The AUROC in discriminating PD vs. PR/SD was 0.95 (95% CI = 0.91–1) (Supplementary Figure S1). The performance of the model is particularly accurate reaching sensitivity of 0.90 (95% CI = 0.68–0.99), specificity of 0.91 (95% CI = 0.78–0.97), PPV of 0.82 (95% CI = 0.60–0.95), and NPV of 0.95 (95% CI = 0.84–0.99).

#### 3.3.2. Progression-Free Survival Prediction

About 65% of patients (108/166) had disease progression during their follow-up period and 40% (67/166) had disease progression within 6 months from the starting of the treatment. The median progression-free survival time was 8 months (95% CI = 7–15 months). Results from univariate Cox regression in the combined datasets and by center are reported in Supplementary Table S4. The analysis of the combined datasets evidenced that patients who were undergoing the second, third, and fourth lines of treatment were at significantly reduced risk of progression compared to those who were performing the first line of treatment (HR < 0.4, *p* < 0.05). Compared to females, males were at significantly higher risk of progression in the PSM dataset (HR = 12.34, *p* = 0.0135) but not in the ICSM (HR = 0.98, *p* = 0.9412) or in the combined datasets (HR = 1.51, *p* = 0.0897). Compared to patients with IHC PDL1 < 1%, patients with IHC PDL1 1–24% (HR = 0.49, *p* = 0.0036), 25–49% (HR = 0.28, *p* = 0.0002), and ≥ 50% (HR = 0.51, *p* = 0.0223) were at significantly lower risk of progression in the combined datasets. Similar risk estimates were observed in ICSM and PSM data except for patients with IHC PDL1 ≥ 50%. Compared to patients with ECOG PS corresponding to 0, those with ECOG PS value of 2 or 3 were at significantly higher risk of progression in the combined datasets (HR = 2.79, *p* = 0.0006) and both in the ICSM (HR = 2.33, *p* = 0.0138) and PSM datasets (HR = 4.80, *p* = 0.0072). Patients affected by anemia (HR = 2.72, *p* < 0.0001) as well as subjects with NLR ≥ 5 (HR = 2.24, *p* < 0.0001) were at higher risk of progression compared to subjects not affected by anemia and with NLR < 5, respectively. Consistent trends of risk were observed both in ICSM (HR for anemia = 2.23, *p* = 0.0009, HR for NLR = 1.92, *p* = 0.0077) and PSM (HR for anemia = 3.86, *p* = 0.0001, HR for NLR = 2.93, *p* = 0.0021) datasets. The presence of pleural metastases was associated with an increased risk of progression in the combined datasets (HR = 1.84, *p* = 0.0382) and in OSM data (HR = 6.06, *p* = 0.0051) with a consistent trend in ICSM data (HR = 1.47, *p* = 0.2442). Patients exposed to oral or intravenous iron supplements were at increased risk of progression in the PSM dataset (HR = 7.39, *p* = 0.0094) as well as patients erythropoietin-exposed that were at higher risk of progression both in the combined datasets (HR = 2.53, *p* = 0.0018) and both in the ICSM (HR = 2.28, *p* = 0.0075) and PSM datasets (HR = 98.64, *p* = 0.0014). Similarly, antibiotic-exposed patients were at significantly high risk of progression in the combined datasets (HR = 2.02, *p* = 0.0272) and in ICSM patients (HR = 2.02, *p* = 0.0354). Antiplatelet/anticoagulant treatments were significantly associated with an increased risk of progression in the combined datasets (HR = 1.79, *p* = 0.0033) and both in the ICSM (HR = 1.64, *p* = 0.0409) and PSM datasets (HR = 2.31, *p* = 0.0151). Lastly, steroid-exposed patients were at increased risk of progression in the combined datasets (HR = 2.30, *p* = 0.0002) and in the ICSM dataset (HR = 2.36, *p* = 0.0004) with consistent trend in the PSM dataset (HR = 1.87, *p* = 0.3037). The multivariate features selection procedure applied to data of 95 ICSM patients without missing values for the set of variables reported in Supplementary Table S1 (43 progression events within 6 months from the starting of the treatment, 70 progression events during the follow-up period) identified IHC PDL1, ECOG PS (binarized: 0–1 versus 2–3), anemia and NLR as the most informative subset of predictors of progression. Cox regression coefficients corresponding to the variables included in the multivariate model fitted on ICSM data are reported in Table 3.

**Figure 1:**
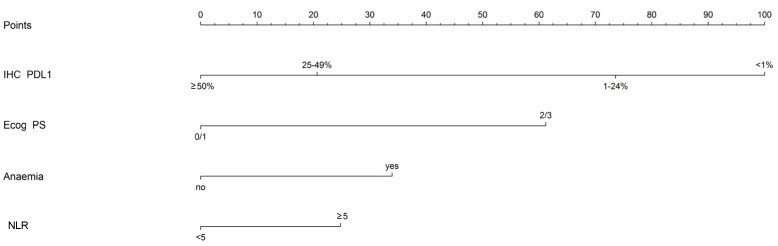
Nomogram for PFS prediction. Each variable’s value is associated with a score (points bar on the upper part of the nomogram, Supplementary Table S4). By summing the points corresponding to the different variables included, it is possible to calculate the subject’s total score.

The nomogram reported in Figure 1 describes graphically the trend and magnitude of the association between the variables included in the multivariate model and the time to progression outcome.

Each variables’ level in the nomogram corresponds to a score value expressed in terms of points: higher scores indicate stronger evidence of association with the outcome. Users can easily compute the total score by summing the partial scores based on the patient’s characteristics (Supplementary Table S5).

When tested on the independent set of 67 patients from PSM with complete information about the set of variables defining the multivariate model (21 progression events within 6 months from the starting of the treatment, 35 progression events during the follow-up period), the AUROC was 0.73 (95% CI = 0.59–0.88) in predicting 6 month PFS, with an overall C statistic of 0.64. Using ICSM data, it has been possible to identify a cut-off value in terms of total points corresponding to a value of 104 that allows discriminating between patients at high risk (i.e., those with a total score ≥104) and low risk (i.e., those with a total score <104) of progression.

**Figure 2:**
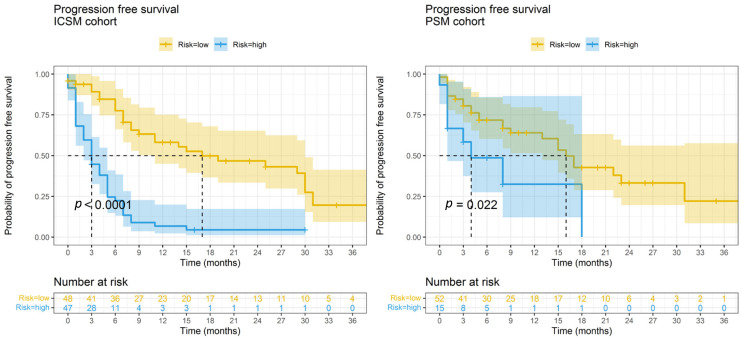
Kaplan–Meier curves of high and low risk of progression patients based on PFS nomograms predictions. Survival profiles of patients at high risk of progression (characterized by total points from the PFS nomogram ≥ 104) and low risk of progression (characterized by total points from the PFS nomogram <104) in the ICSM and OSM datasets, respectively. *p* = *p*-value from the log-rank test. Graphical representation was truncated at 36 months.

Kaplan–Meier curves reported in Figure 2 confirm that the survival profiles of high and low-risk patients are significantly different both in the ICSM and OSM datasets (log-rank *p* < 0.03), with trends of risk consistent between centers.

High-risk patients from ICSM are characterized by a median PFS time of 3 months (95% CI = 2–5) and a probability of 6-month PFS of 0.22 (95% CI = 0.13–0.38), while low-risk patients had a median survival time of 17 months (95% CI = 11–30) by a probability of 6-month PFS of 0.77 (95% CI = 0.66–0.91). When tested on PSM data, the median survival time for high-risk patients was 4 months (95% CI = 1-NA), with a probability of 6 month PFS of 0.49 (95% CI = 0.27–0.86), and the median survival time for low-risk patients was 16 months (95% CI = 13-NA), with a probability of 6 month PFS of 0.72 (95% CI = 0.60–0.86).

The calibration plot on PSM data is reported in Supplementary Figure S2, showing a suitable agreement between predicted and observed progression probabilities when predicting 6-month PFS for low-risk patients while lower performances for high-risk patients.

#### 3.3.3. Overall Survival Prediction

About 57% of patients (94/166) died during their follow-up period: 26% (43/166) and 34% (56/166) died within 6 and 12 months from the starting of the treatment, respectively. The median overall survival time was 17 months (95% CI = 15–24). Results from univariate Cox regression evaluating the association between candidate variables of interest and the time to death outcome by center and in the combined datasets are reported in Supplementary Table S6. The risk of death was inversely proportional to the treatment line: when focusing on the combined datasets it was possible to observe that patients undergoing second (HR = 0.36, *p* = 0.0006), third (HR = 0.36, *p* = 0.0063), and fourth treatment line (HR = 0.33, *p* = 0.0048) were at significantly lower risk of death compared to first treatment line patients. This trend of risk was similar between ICSM and PSM datasets. Male sex was statistically associated with an increased risk of death only in the PSM dataset (HR = 8.72, *p* = 0.0341). Former and current smokers were at significantly lower risk of death compared to never smokers in the PSM dataset (HR = 0.27, *p* = 0.0428) but not in ICSM data (HR = 1.30, *p* = 0.6614) and in the combined cohort (HR = 0.76, *p* = 0.5271). When compared with patients with IHC PDL1 < 1%, patients with IHC PDL1 ranging 1–24% (HR = 0.48, *p* = 0.0065), 25–49% (HR = 0.30, *p* = 0.0011), and ≥ 50% (HR = 0.64, *p* = 0.1761) were at lower risk of death in combined datasets. A similar and more evident trend was observed from the analysis of ICSM data, with patients with IHC PDL1 ranging 1–24% (HR = 0.43, *p* = 0.0031), 25–49% (HR = 0.24, *p* = 0.0013), and ≥ 50% (HR = 0.29, *p* = 0.0408) at lower risk compared to patients with IHC PDL1 < 1%. In the combined datasets analysis, patients with ECOG PS corresponding to 2 or 3 were at significantly higher risk of death compared to patients characterized by values corresponding to 0 (HR = 3.48, *p* < 0.0001): this association was consistent across datasets. Anemia was associated with a statistically significant increase in terms of probability of relapse in ICSM data (HR = 2.66, *p* = 0.0002), PSM data (HR = 4.18, *p* = 0.0005) and in the combined datasets (HR = 3.02, *p* < 0.0001). Moreover, NLR ≥ 5 was also associated with an increased probability of progression both in ICSM (HR = 2.14, *p* = 0.0035), PSM data (HR = 2.8, *p* = 0.0103) and in the combined datasets (HR = 2.30, *p* = 0.0001). LDH ≥ 325 was associated with an increased risk of progression in ICSM data only (HR = 1.81, *p* = 0.0249).

The presence of pleural metastases was associated with a statistically significant increase in terms of death risk in PSM data (HR = 6.98, *p* = 0.0032) with consistent trend of association in ICSM data (HR = 1.68, *p* = 0.1230) and in the two combined datasets (HR = 2.06, *p* = 0.0157). A similar trend was observed in the presence of a pool of low-frequency metastasis sites, being associated with a significantly increased risk of progression in PSM data (HR = 2.29, *p* = 0.0397), with a consistent effect in ICSM data (HR = 1.33, *p* = 0.3500) and in combined datasets (HR = 1.62, *p* = 0.0454).

Erythropoietin-exposed patients were at increased risk of death in ICSM data (HR = 2.67, *p* = 0.0019), PSM data (HR = 69.5, *p* = 0.0027) and in combined datasets (HR = 2.96, *p* = 0.0004) as well as patients exposed to antibiotic that were at increased risk of death in ICSM data (HR = 2.36, *p* = 0.0116), with a consistent trend of risk in PSM data (HR = 5.66, *p* = 0.1065) and a confirmed association in combined datasets (HR = 2.48, *p* = 0.0050). Oral or intravenous iron supplements were also associated with a statistically significant increase in terms of risk of death in ICSM data (HR = 1.78, *p* = 0.0231), PSM data (HR = 2.30, *p* = 0.0338) and in combined datasets (HR = 1.91, *p* = 0.0025). Lastly, steroid-exposed patients were at increased risk of death in ICSM data (HR = 2.11, *p* = 0.0029) and in combined datasets (HR = 2.1, *p* = 0.0017).

As previously described for the PFS outcome, a multivariate features selection process was performed on data from 95 patients from ICSM without missing values for the set of variables reported in Supplementary Table S1 (29 and 39 death events within 6 and 12 months from the starting of the treatment, respectively, while 67 death events during the entire follow-up period) identified IHC PDL1, ECOG PS (binarized), anemia and NLR as the most informative subset of predictors of death. Cox regression coefficients corresponding to the variables included in the multivariate model fitted on ICSM data are reported in Table 4.

**Figure 3:**
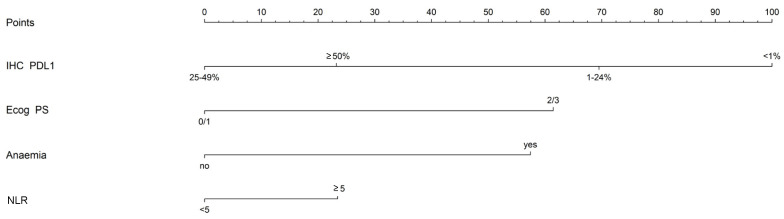
Nomogram for OS prediction. Each variable’s value is associated with a score (points bar on the upper part of the nomogram, Supplementary Table S4). By summing the points corresponding to the different variables included, it is possible to calculate the subject’s total score.

The nomogram reported in Figure 3 describes graphically the magnitude of the association between the variables defining the multivariate model and the time to death outcome. Users can compute the total points based on patients’ characteristics using the information reported in Supplementary Table S4.

When tested on data from the independent set of 67 patients from PSM with complete information about the set of variables defining the multivariate model (14 and 17 death events within 6 and 12 months from the starting of the treatment, respectively, 27 death events during the entire follow-up period) the AUROC in predicting 6- and 12-month OS was 0.82 (95% CI = 0.70–0.95) and 0.81 (95% CI = 0.67–0.95) respectively, with an overall C statistic of 0.66.

The cut-off value in terms of total score identified using ICSM data was 87, discriminating between patients at high risk (i.e., those with a total score ≥87) and low risk (i.e., those with a total score <87) of death.

**Figure 4:**
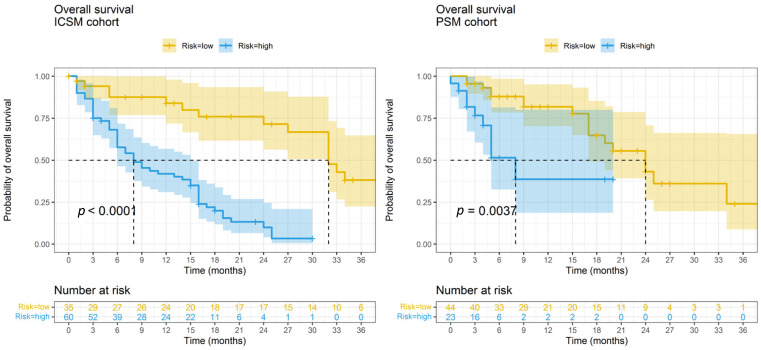
Kaplan–Meier curves of high and low risk of death patients based on OS nomograms predictions. Survival profiles of patients at high risk of death (characterized by total points from the OS nomogram ≥ 87) and low risk of progression (characterized by total points from the OS nomogram < 87) in the ICSM and OSM datasets, respectively. *p* = *p*-value from the log-rank test. Graphical representation was truncated at 36 months.

Kaplan–Meier curves reported in Figure 4 confirm that the OS profiles of high and low-risk patients are significantly different both in the ICSM and OSM datasets (log-rank *p* < 0.01), with trends of risk consistent between centers. In the ICSM dataset, high-risk patients are characterized by a median survival time of 8 months (95% CI = 6–15) and probability of 6- and 12-month OS of 0.58 (95% CI = 0.46–0.72) and 0.42 (95% CI = 0.31–0.57) while low-risk patients by a median survival time of 32 months (95% CI = 32-NA) and probability of 6- and 12-month OS of 0.87 (95% CI = 0.77–1) and 0.84 (95% CI = 0.72–0.98), respectively.

When tested on PSM data the median survival time of high-risk patients was 8 months (95% CI = 5-NA) and the probability of 6- and 12-month OS was 0.51 (95% CI = 0.32–0.81) and 0.39 (95% CI = 0.19–0.80). Low-risk patients from PSM were characterized by a median survival time of 24 months (95% CI = 17-NA) with a 6- and 12-month OS probability of 0.88 (95% CI = 0.78–0.98) and 0.82 (95% CI = 0.70–0.95). The calibration plot on PSM data is reported in Supplementary Figure S3, showing a high degree of agreement between predicted and observed survival probabilities when predicting 6 and 12 months OS for low-risk and high-risk patients.

## 4. Discussion

The presented work included 166 consecutive patients treated at two second-level specialized cancer centers. These patients, unselected for comorbidities and demographic characteristics, represent the composition of a population treated in a “real world” setting.

Several real-life works have been published regarding patients with advanced NSCLC treated with ICI. Among all, two studies deriving from the Italian Extended Access Program of nivolumab represent the most extensive clinical experience with nivolumab outside randomized clinical trials [13,14]. The first study included 371 patients with lung SCC and confirmed the activity and efficacy of the drug in a real-life context, with an acceptable safety profile; the presence of liver and bone metastases, as well as an ECOG PS > 0, appeared to negatively affect OS. The second study included 1588 pre-treated patients with advanced NSCLC of non-squamous cell histotype. In the pre-planned subgroup analyses, notably, elderly patients appeared to have similar responses to the general study population, and those with brain metastases had a median OS of 8.6 months, with a 1-year survival rate of 43%.

The population analyzed in the present study includes mostly male subjects (75%) aged ≥ 65 years (70%) and former or active smokers (90%). These data reflect the higher incidence of lung cancer in the elderly and male population. Both of these characteristics are associated with a higher prevalence of comorbidities [15,16,17]. All patients received immunotherapy as monotherapy, in accordance with the indications in force at the time of the study. Furthermore, the aim of the study was to evaluate the relationship between clinical-biological variables and the efficacy of immunotherapy alone, removing possible confounding factors derived from concomitant chemotherapy [18].

The detrimental role of anemia at the beginning of ICI represents the most innovative finding of this study. About 50% of patients presented with anemia, mostly linked to a chronic disease condition (70%) and only in a minority of cases to iron deficiency or to previous chemotherapy treatments. Anemia is the most common hematological manifestation in cancer patients, being present in more than 30% of cases at diagnosis, and is independently associated with shorter survival [19,20,21]. Several studies have also shown how cancer-related anemia is associated with reduced efficacy of chemotherapy, radiotherapy, and combination treatments [21,22,23,24,25], as well as with an impaired quality of life [26]. It is more common in patients with advanced disease, where it represents a consequence of chronic inflammation or of the direct infiltration of the bone marrow. Cancer-related anemia is characterized by biological and hematological aspects that resemble those described in chronic inflammatory disease. Furthermore, impaired nutritional status can induce or contribute to cancer anemia and be related to a significant decline in PS and quality of life, with progressive worsening of cognitive functions and activity levels [27]. The results here presented indicate that the presence of anemia at treatment start has a negative impact on the probability of obtaining a response to ICI, on the probability of disease progression at 6 months, and on the probability of death at 6 and 12 months. To confirm this, in the elaborated nomogram, anemia was the most important factor in terms of the probability of progression at 6 months and death at 6 and 12 months.

Also, the NLR measured before starting the treatment has an important predictive and prognostic value influencing the probability of obtaining a response to ICI, the probability of disease progression at 6 months, and the probability of death at 6 and 12 months. Moreover, NLR resulted among the first four parameters influencing the prognosis in the nomogram. It is well known that high NLR at baseline has negative prognostic role in patients treated with ICI [28]. Recent studies have described that increased circulating neutrophils, the numerator of NLR, are directly linked to the number of intratumoral neutrophilic populations, which may have the potential to compromise the antitumor immune response, and that a low absolute lymphocyte count can reflect damage to cell-mediated immunity. Moreover, neutrophils are involved in tumor initiation, progression, and metastatization by direct effect or affecting cells of the tumor microenvironment. This effect is achieved through the secretion and release of various chemokines and cytokines, including transforming growth factor beta, vascular endothelial growth factor, IL-6, IL-8, and matrix metalloproteinases. Finally, recent studies have also shown an inverse relationship between elevated values of circulating neutrophils and CD8+ tumor-suppressing T cell infiltrating lung cancer [29].

In the present study, about 40% of patients had an ECOG PS = 0 before treatment start, about 40% ECOG PS = 1, and about 10% an ECOG PS = 2–3. ECOG PS influenced the probability of disease progression at 6 months, the probability of death at 6 and 12 months, and ranks among the four most important variables in the nomogram. PS is a subjective composite measure used by clinicians to measure functional capacity and the likelihood of AEs, quality of life, and survival after treatment. Most clinical trials purposely excluded patients with poor PS, and existing evidence of limited benefits among poor PS patients derives from only a few small trials [30,31]. Due to better-perceived tolerance of ICIs compared with cytotoxic chemotherapy, results from the randomized phase III clinical trials have been extended to patients with PS scores of 2 or higher, leading to liberal use of ICIs in real-life patients. A recent study included 74 patients with an ECOG PS score of at least 2 at the start of pembrolizumab, confirming it as an independent risk factor for worse PFS (HR, 2.02) and OS [32]. Petrillo et al. reported a median OS of 4.5 months in a similar cohort of patients with ECOG PS scores of at least 2 [33]. Therapeutic decisions for patients with moderate to poor PS remain tricky due dynamic nature of this measure, depending not only on patients’ intrinsic characteristics but also on the symptoms of the cancer disease itself, which could be likely to benefit from an appropriate treatment. For this reason, it is imperative to include objective and dynamic measurements of functional status in future clinical trials to facilitate the identification of patients with borderline PS who could achieve potential clinical benefit and improvement in quality of life from ICIs.

In this study, about 25% of patients had an IHC expression of PD-L1 on a tumor tissue <1%, about 30% between 1% and 24%, about 25% between 25% and 49%, while about 30% had an expression ≥ 50%. Although the expression of PD-L1 is neither necessary nor sufficient to define the possibility of response to immunotherapy, it may indicate a greater probability of response to immunotherapy [34,35]. In this study, the lower expression of PD-L1 influenced the probability of response, as well as the probability of having a progression at 6 months or death at 6 and 12 months. The expression of PD-L1 was also placed among the four most important variables in the nomogram.

In our study, LDH measured before treatment initiation predicted the probability of response to ICI but did not influence PFS and OS. It is well known that patients with elevated LDH have less probability of response to treatments with ICIs since high LDH is an expression of an increased anaerobic glycolytic activity of the tumor and of hypoxia-dependent tumor necrosis [36]. In hypoxic tissues, the function of immune cells can be hampered by glucose deprivation or by the acidic microenvironment. In fact, metabolic interplay and nutrient competition between cancer cells and T cells exist and are recognized as key drivers of carcinogenesis. The increased glucose addition and glycolysis rate of rapidly growing cancer cells (Warburg effect) consume most nutrients from the microenvironment. As a consequence, the tumor-imposed metabolic restrictions reduce T cell responsiveness [37,38]. T cells became unable to produce cytokines and to develop into tumor-specific T effector cells, leading to a state of anergy [39]. Glucose deprivation can prevent tumor-infiltrating CD8+ cells from functioning by altering interferon gamma production. Similarly, an increase in glucose uptake and lactate production has been evidenced in naive B cells after stimulation. Moreover, in these conditions, macrophages can polarize toward an anti-inflammatory phenotype with pro-tumoral properties, and natural killer (NK) can diminish cytotoxic activity due to an impairment of glucose metabolism and disruption of mTOR signaling.

Few and discordant studies have specifically evaluated the prognostic value of comorbidities in patients with advanced LC [40,41,42]. However, these studies did not evaluate patients receiving ICIs. In the current epidemiological context characterized by an aging population and the consequent increase in the prevalence of chronic degenerative diseases, the phenomenon of multimorbidity is common in clinical practice and can significantly modify both the clinical expression of index disease and the response to prescribed treatments. In the present study, ACCI was selected among the variables influencing the probability of response, but it did not seem to influence the probability of progression or death.

Regarding irAEs, several studies have identified a frequency of 70% of side effects, with a time of onset between 3 and 6 months from the start of therapy [43,44,45]. In this study, the frequency of AEs was low, with only 31% of patients experiencing at least one AE; however, as reported in the literature, asthenia was the most frequent AE, followed by gastrointestinal toxicity and endocrine side effects. Discontinuation of the treatment due to AEs was rare, occurring in only four patients (2.5%).

The use of concomitant drugs could impact the possibility of response to immunotherapy. While the main perspective trials excluded patients taking more than 10 mg of prednisone or equivalent, retrospective studies have evaluated possible interference between immunotherapy and corticosteroid in real life, particularly in patients who had taken them before ICI; the results appear to be conflicting since some of them showed a decrease in PFS and objective responses, while others did not find such interference [46,47,48,49,50,51]. Antibiotic therapy was also correlated with a negative outcome in patients treated with ICIs alone in terms of overall response, time to progression, and OS [52,53]. The pathophysiology appears to be related to the deregulation of intestinal microbiota, which is detrimental to ICI response. As for PPIs, the results are scarce and contradictory; however, a recent study showed a lower response to immunotherapy treatments in patients treated with this drug class [54]. In the study, the authors carried out an unplanned analysis of the data from the OAK and POPLAR trials to verify the impact of the use of PPIs and antibiotics in patients with lung cancer receiving ICI. In multivariate analysis, the use of PPIs was associated with a reduced OS and PFS. However, the two drugs used did not alter the expected outcomes in docetaxel-treated patients. The analysis of the data of our study showed that the use of antibiotics and steroids influences PFS and OS but not the probability of response, and no correlations were found with PPIs use.

The effects of anticoagulants in modulating the innate immunity, affecting the antibacterial immune response, are well known, but few data are available regarding patients treated with ICI [55]. Wang and colleagues evaluated 330 patients with melanoma treated with PD-1 inhibitors without reporting an association between disease response, disease progression, OS, and use of non-steroidal anti-inflammatory drugs (including aspirin) [56]. In the present study, the impact of antiplatelet/anticoagulant treatments was evaluated, and, at univariate analysis, they seem to increase the probability of PD with an impact on PFS but without influence on OS.

Finally, the present work provides a nomogram with a visual method to predict response, progression at 6 months, and death at 6 and 12 months from immunotherapy start that may be useful to tailor treatments according to patient characteristics and easily usable in daily clinical practice. The strength of this nomogram resides in the prompt availability of the selected variables, where other available instruments chose indicators of limited use in clinical practice. In particular, anemia was never included among the variables analyzed [57,58]. To date, a recent work reported a prognostic score for ICI in patients with advanced lung cancer (EPSILoN); the selected variables were ECOG PS, smoker status, presence of liver metastases, LDH, and NLR value. The combination of these variables divides patients into three groups with different prognoses [59].

In the era of personalized medicine, the results of this study are useful for stratifying the patients and tailoring the treatments, considering both the histological findings and the clinical features of the patients. Due to the fast development of new systemic drugs and new drug combinations for advanced NSCLC treatment in the next future, the results will be useful to help clinicians to better select appropriate treatment decisions and design dedicated clinical trials for frail or comorbid patients. We acknowledge limitations in our analysis, including the small sample size that may limit the power of the statistical analysis and the absence of a calculation of the sample size; the lack of centralized imaging review; the inclusion of only two university oncologic centers in Northern Italy; and the choice of physician-assessed response. So, further efforts on a multicenter study and prospective data collection, including other potential factors, are encouraged to refine the nomogram.

## 5. Conclusions

This study is a real-life analysis of the clinical factors associated with the efficacy of anti-PD-1 and anti-PDL-1 ICI in patients affected by advanced-stage NSCLC. The results emphasize how the global clinical setting has to be taken into account, as basic clinical parameters have an impact on the response to therapy. In fact, the patient’s PS, LDH, NLR, and the presence of anemia before the initiation of immunotherapy have been shown to predict the efficacy of immunotherapy beyond histotype and tumor expression of PD-L1. As clinical trials usually consider selected patients without comorbidities, this study provides a more comprehensive insight into the daily clinical practice manifestations involved in response to immunotherapies.

## Figures and Tables

**Table 1:** Demographic and clinical characteristics of the analyzed cohorts. Variable = analyzed variable; Value = value that each categorical variable may assume; Overall = absolute and relative frequency (%) of categorical variables in the overall cohort; ICSM = absolute and relative frequency (%) of categorical variables in the ICSM cohort; PSM = absolute and relative frequency (%) of categorical variables in the PSM cohort; *p* = *p*-value from the Pearson chi-square test for independence between considered variables and cohorts (ICSM/PSM). ^#^ Including: soft tissues and adrenal glands.

	Overall	ICSM	PSM
Variable	*n* = 166	*n* = 95	*n* = 71
**Age at treatment start (years)**			
<65	54 (32.53%)	30 (31.58%)	24 (33.8%)
≥65	112 (67.47%)	65 (68.42%)	47 (66.2%)
**Sex**			
Females	37 (22.29%)	25 (26.32%)	12 (16.9%)
Males	129 (77.71%)	70 (73.68%)	59 (83.1%)
**Smoker habits**			
Never	12 (7.23%)	6 (6.32%)	6 (8.45%)
Former	80 (48.19%)	22 (23.16%)	58 (81.69%)
Active	74 (44.58%)	67 (70.53%)	7 (9.86%)
**Histotype**			
ADC	124 (74.7%)	67 (70.53%)	57 (80.28%)
SCC	42 (25.3%)	28 (29.47%)	14 (19.72%)
**Line of treatment**			
1	34 (20.48%)	8 (8.42%)	26 (36.62%)
2	95 (57.23%)	62 (65.26%)	33 (46.48%)
3	23 (13.86%)	15 (15.79%)	8 (11.27%)
4	14 (8.43%)	10 (10.53%)	4 (5.63%)
**Immunotherapy treatment**			
Atezolizumab	26 (15.66%)	8 (8.43%)	18 (25.35%)
Nivolumab	84 (50.6%)	70 (73.68%)	14 (19.72%)
Pembrolizumab	56 (33.73%)	17 (17.89%)	39 (54.93%)
**IHC PDL1 (%)**			
<1%	44 (27.16%)	37 (38.95%)	7 (10.45%)
1–24%	46 (28.4%)	35 (36.84%)	11 (16.42%)
25–49%	28 (17.28%)	15 (15.79%)	13 (19.4%)
≥50%	44 (27.16%)	8 (8.42%)	36 (53.73%)
**Disease stage**			
IIIA	3 (1.81%)	0 (0%)	3 (4.23%)
IIIB	28 (16.87%)	17 (17.89%)	11 (15.49%)
IIIC	8 (4.82%)	3 (3.16%)	5 (7.04%)
IV	127 (76.51%)	75 (78.95%)	52 (73.24%)
**Lung metastasis**			
No	20 (12.05%)	20 (21.05%)	0 (0%)
Yes	146 (87.95%)	75 (78.95%)	71 (100%)
**Liver metastasis**			
No	148 (89.16%)	80 (84.21%)	68 (95.77%)
Yes	18 (10.84%)	15 (15.79%)	3 (4.23%)
**Lymph nodes metastasis**			
No	21 (12.65%)	11 (11.58%)	10 (14.08%)
Yes	145 (87.35%)	84 (88.42%)	61 (85.92%)
**Bone metastasis**			
No	128 (77.11%)	65 (68.42%)	63 (88.73%)
Yes	38 (22.89%)	30 (31.58%)	8 (11.27%)
**Brain metastasis**			
No	145 (87.35%)	86 (90.53%)	59 (83.1%)
Yes	21 (12.65%)	9 (9.47%)	12 (16.9%)
**Pleural metastasis**			
No	151 (90.96%)	83 (87.37%)	68 (95.77%)
Yes	15 (9.04%)	12 (12.63%)	3 (4.23%)
**Other metastasis #**			
No	129 (77.71%)	75 (78.95%)	54 (76.06%)
Yes	37 (22.29%)	20 (21.05%)	17 (23.94%)
**ECOG PS**			
0	75 (45.18%)	40 (42.11%)	35 (49.3%)
1	72 (43.37%)	42 (44.21%)	30 (42.25%)
2	16 (9.64%)	10 (10.53%)	6 (8.45%)
3	3 (1.81%)	3 (3.16%)	0 (0%)
**LDH (mU/mL)**			
<325	71 (43.56%)	36 (37.89%)	35 (51.47%)
≥325	92 (56.44%)	59 (62.11%)	33 (48.53%)
**NLR**			
<5	81 (48.8%)	40 (42.11%)	41 (57.75%)
≥5	85 (51.2%)	55 (57.89%)	30 (42.25%)
**Anemia**			
No	89 (53.61%)	44 (46.32%)	45 (63.38%)
Yes	77 (46.39%)	51 (53.68%)	26 (36.62%)
**Causes of anemia**			
Chronic disease	55 (71.43%)	34 (66.67%)	21 (80.77%)
Sideropenic	9 (11.69%)	4 (7.84%)	5 (19.23%)
Previous CT toxicity	13 (16.88%)	13 (25.49%)	0 (0%)
**Thrombosis before ICI**			
No	132 (79.52%)	63 (66.32%)	69 (97.18%)
Yes	34 (20.48%)	32 (33.68%)	2 (2.82%)
**ACCI (points)**			
<9	56 (33.73%)	45 (47.37%)	11 (15.49%)
≥9	110 (66.27%)	50 (52.63%)	60 (84.51%)
**ICI toxicity**			
No	113 (68.48%)	49 (52.13%)	64 (90.14%)
Yes	52 (31.52%)	45 (47.87%)	7 (9.86%)
**Blood transfusions**			
No	155 (93.37%)	87 (91.58%)	68 (95.77%)
Yes	11 (6.63%)	8 (8.42%)	3 (4.23%)
**Oral or intravenous iron supplements**			
No	158 (95.18%)	89 (93.68%)	69 (97.18%)
Yes	8 (4.82%)	6 (6.32%)	2 (2.82%)
**Erythropoietin use**			
No	150 (90.36%)	80 (84.21%)	70 (98.59%)
Yes	16 (9.64%)	15 (15.79%)	1 (1.41%)
**Antibiotic use**			
No	148 (90.24%)	80 (86.02%)	68 (95.77%)
Yes	16 (9.76%)	13 (13.98%)	3 (4.23%)
**Proton pump inhibitor use**			
No	59 (35.54%)	31 (32.63%)	28 (39.44%)
Yes	107 (64.46%)	64 (67.37%)	43 (60.56%)
**Antiplatelet/anticoagulant treatment**			
No	83 (50%)	43 (45.26%)	40 (56.34%)
Yes	83 (50%)	52 (54.74%)	31 (43.66%)
**Steroid use**			
No	122 (73.49%)	56 (58.95%)	66 (92.96%)
Yes	44 (26.51%)	39 (41.05%)	5 (7.04%)

Abbreviations: ACCI, Age-adjusted Charlson Comorbidity Index; ADC, adenocarcinoma; CT, chemotherapy; ECOG PS, Eastern Cooperative Oncology Group Performance Status; ICI, immune checkpoint inhibitor; ICSM, Istituti Clinici Scientifici Maugeri; IHC, immunohistochemical; NLR, neutrophil/lymphocyte ratio; PD, progressive disease; PR, partial response; PSM, Policlinico San Matteo; SCC, squamocellular; SD, stable disease.

**Table 2:** Association between variables of interest and best response. Variable = analyzed variable and corresponding values; PD = absolute and relative frequency (%) of patients by variable’s category among those with best response type PD; PR = absolute and relative frequency (%) of patients by variable’s category among those with best response type PR; SD = absolute and relative frequency (%) of patients by variable’s category among those with best response type SD; * *p* < 0.05; ^#^ Including soft tissues and adrenal glands.

				PD vs. PR/SD	
Variable	PD	PR	SD	OR (95% CI)	*p*
**Line of treatment**					
1st	15 (23.81%)	11 (25%)	8 (13.56%)	Baseline	
2nd	31 (49.21%)	22 (50%)	42 (71.19%)	0.38 (0.15–0.94)	0.0383
3rd	12 (19.05%)	5 (11.36%)	6 (10.17%)	0.91 (0.29–2.82)	0.8643
4th	5 (7.94%)	6 (13.64%)	3 (5.08%)	0.42 (0.10–1.61)	0.2146
**Sex**					
Females	14 (22.22%)	8 (18.18%)	15 (25.42%)	Baseline	
Males	49 (77.78%)	36 (81.82%)	44 (74.58%)	1.11 (0.52–2.45)	0.7862
**Age at treatment start (years)**					
<65	19 (30.16%)	15 (34.09%)	20 (33.9%)	Baseline	
≥65	44 (69.84%)	29 (65.91%)	39 (66.1%)	1.18 (0.60–2.36)	0.6421
**Smoking habits**					
Never	6 (9.52%)	2 (4.55%)	4 (6.78%)	Baseline	
Former/active	57 (90.48%)	42 (95.45%)	55 (93.22%)	0.54 (0.16–1.85)	0.3193
**Histotype**					
ADC	52 (82.54%)	37 (84.09%)	35 (59.32%)	Baseline	
SCC	11 (17.46%)	7 (15.91%)	24 (40.68%)	0.43 (0.19–0.93)	0.0384
**IHC PDL1**					
<1%	29 (46.03%)	6 (13.64%)	9 (16.36%)	Baseline	
1–24%	13 (20.63%)	15 (34.09%)	18 (32.73%)	0.21 (0.08–0.50)	0.0006
25–49%	8 (12.7%)	8 (18.18%)	12 (21.82%)	0.23 (0.08–0.64)	0.0060
≥50%	13 (20.63%)	15 (34.09%)	16 (29.09%)	0.27 (0.09–0.76)	0.0139
**ECOG PS**					
0	24 (38.1%)	21 (47.73%)	30 (50.85%)	Baseline	
1	29 (46.03%)	21 (47.73%)	22 (37.29%)	1.40 (0.70–2.79)	0.3392
2–3	10 (15.87%)	2 (4.55%)	7 (11.86%)	2.17 (0.77–6.25)	0.1431
**Anemia**					
No	8 (12.7%)	37 (84.09%)	44 (74.58%)	Baseline	
Yes	55 (87.3%)	7 (15.91%)	15 (25.42%)	24.16 (10.49–62.15)	<0.0001
**NLR**					
<5	10 (15.87%)	34 (77.27%)	37 (62.71%)	Baseline	
≥5	53 (84.13%)	10 (22.73%)	22 (37.29%)	11.21 (5.22–26.09)	<0.0001
**LDH (mU/mL)**					
<325	4 (6.35%)	30 (69.77%)	37 (64.91%)	Baseline	
≥325	59 (93.65%)	13 (30.23%)	20 (35.09%)	29.01 (10.76–101.96)	<0.0001
**Lung metastasis**					
No	8 (12.7%)	4 (9.09%)	8 (13.56%)	Baseline	
Yes	55 (87.3%)	40 (90.91%)	51 (86.44%)	1.31 (0.49–3.7)	0.5952
**Liver metastasis**					
No	52 (82.54%)	39 (88.64%)	57 (96.61%)	Baseline	
Yes	11 (17.46%)	5 (11.36%)	2 (3.39%)	2.45 (0.89–7.13)	0.0870
**Lymph nodes metastasis**					
No	11 (17.46%)	5 (11.36%)	5 (8.47%)	Baseline	
Yes	52 (82.54%)	39 (88.64%)	54 (91.53%)	0.47 (0.18–1.22)	0.1210
**Bone metastasis**					
No	43 (68.25%)	34 (77.27%)	51 (86.44%)	Baseline	
Yes	20 (31.75%)	10 (22.73%)	8 (13.56%)	1.87 (0.88–4.02)	0.1049
**Brain metastasis**					
No	54 (85.71%)	40 (90.91%)	51 (86.44%)	Baseline	
Yes	9 (14.29%)	4 (9.09%)	8 (13.56%)	1.45 (0.55–3.77)	0.4433
**Pleural metastasis**					
No	51 (80.95%)	43 (97.73%)	57 (96.61%)	Baseline	
Yes	12 (19.05%)	1 (2.27%)	2 (3.39%)	6.95 (2.07–31.75)	0.0040
**Other metastasis ^#^**					
No	46 (73.02%)	34 (77.27%)	49 (83.05%)	Baseline	
Yes	17 (26.98%)	10 (22.73%)	10 (16.95%)	1.61 (0.75–3.43)	0.2184
**Thrombosis before therapy**					
No	41 (65.08%)	39 (88.64%)	52 (88.14%)	Baseline	
Yes	22 (34.92%)	5 (11.36%)	7 (11.86%)	3.46 (1.5–8.32)	0.0043
**ACCI (points)**					
<9	20 (31.75%)	21 (47.73%)	15 (25.42%)	Baseline	
≥9	43 (68.25%)	23 (52.27%)	44 (74.58%)	1.58 (0.78–3.30)	0.2142
**Toxicity from ICI**					
No	44 (70.97%)	27 (61.36%)	42 (71.19%)	Baseline	
Yes	18 (29.03%)	17 (38.64%)	17 (28.81%)	0.55 (0.25–1.17)	0.1268
**Blood transfusions**					
No	57 (90.48%)	41 (93.18%)	57 (96.61%)	Baseline	
Yes	6 (9.52%)	3 (6.82%)	2 (3.39%)	1.87 (0.53–6.85)	0.3276
**Oral or intravenous iron supplements**					
No	59 (93.65%)	43 (97.73%)	56 (94.92%)	Baseline	
Yes	4 (6.35%)	1 (2.27%)	3 (5.08%)	1.48 (0.33–6.62)	0.5915
**Erythropoitin use**					
No	56 (88.89%)	43 (97.73%)	51 (86.44%)	Baseline	
Yes	7 (11.11%)	1 (2.27%)	8 (13.56%)	0.98 (0.32–2.86)	0.9689
**Antibiotic use**					
No	55 (90.16%)	40 (90.91%)	53 (89.83%)	Baseline	
Yes	6 (9.84%)	4 (9.09%)	6 (10.17%)	0.84 (0.27–2.44)	0.7527
**Proton pump inhibitor use**					
No	24 (38.1%)	16 (36.36%)	19 (32.2%)	Baseline	
Yes	39 (61.9%)	28 (63.64%)	40 (67.8%)	0.79 (0.4–1.53)	0.4784
**Antiplatelet/anticoagulant treatment**					
No	24 (38.1%)	24 (54.55%)	35 (59.32%)	Baseline	
Yes	39 (61.9%)	20 (45.45%)	24 (40.68%)	2.06 (1.08–3.99)	0.0289 *
**Type of ICI**					
Atezolizumab	6 (9.52%)	3 (6.82%)	17 (28,81%)	Baseline	
Nivolumab	33 (52.38%)	23 (52.27%)	28 (47.46%)	0.38 (0.06–2.35)	0.2836
Pembrolizumab	24 (38.1%)	18 (40.91%)	14 (23.73%)	0.76 (0.13–4.63)	0.7577
**Steroid use**					
No	40 (63.49%)	34 (77.27%)	48 (81.36%)	Baseline	
Yes	23 (36.51%)	10 (22.73%)	11 (18.64%)	1.8 (0.84–3.85)	0.1280

Abbreviations: ADC, adenocarcinoma; ECOG PS, Eastern Cooperative Oncology Group Performance Status; IHC, immunohistochemical; CIRS, Cumulative Illness Rating Scale; NLR, neutrophil/lymphocyte ratio; SCC, squamocellular carcinoma; PD, progressive disease; PR, partial response; SD, stable disease.

**Table 3:** Results from multivariate Cox proportional hazard regression model for time to progression outcome. HR (95% CI) = hazard ratio and 95% confidence interval; *p* = *p*-value. * *p* < 0.05.

	ICSM		PSM	
Variable	HR (95% CI)	*p*	HR (95% CI)	*p*
IHC PDL1: 1–24%	1.65 (1.37–2.13)	0.127	1.49 (0.29–7.56)	0.633
IHC PDL1: 25–49%	0.27 (0.12–0.65)	0.003 *	0.79 (0.18–3.40)	0.749
IHC PDL1: ≥50%	0.19 (0.06–0.66)	0.008 *	0.26 (0.61–8.39)	0.223
ECOG PS: 2/3	2.73 (1.38–5.37)	0.003 *	1.60 (0.51–5.07)	0.423
Anemia: yes	1.74 (0.97–3.12)	0.060	1.96 (0.88–4.40)	0.101
NLR ≥ 5	1.50 (0.85–2.66)	0.164	2.71 (1.09–6.75)	0.031 *

**Table 4:** Results from multivariate Cox proportional hazard regression model for time to death outcome. HR (95% CI) = hazard ratio and 95% confidence interval; *p* = *p*-value. * *p* < 0.05.

	ICSM		PSM	
Variable	HR (95% CI)	*p*	HR (95% CI)	*p*
IHC PDL1: 1–24%	1.67 (1.37–2.19)	0.172	1.87 (1.45–3.56)	0.199
IHC PDL1: 25–49%	0.27 (0.10–0.67)	0.005 *	0.36 (0.24–0.76)	0.189
IHC PDL1: ≥50%	0.36 (0.11–1.21)	0.098	0.46 (0.24–0.87)	0.099
ECOG PS: 2/3	2.26 (1.16–4.40)	0.016 *	3.03 (0.89–10.26)	0.075
Anemia: yes	2.14 (1.12–4.09)	0.021 *	1.94 (0.72–5.24)	0.190
NLR ≥ 5	1.36 (0.71–2.61)	0.346	2.88 (0.97–8.56)	0.057

## Data Availability

Not applicable.

## Supplementary Materials

The following supporting information can be downloaded at: https://www.mdpi.com/article/10.3390/jpm12050679/s1, Supplementary Figure S1: ROC curve for response type prediction on PSM data; Supplementary Figure S2: Calibration plot on PSM data corresponding to the progression risk categories defined on ICSM data. The plot represents graphically the observed and predicted probabilities of progression-free survival for patients belonging to the low- and high-risk categories. The dashed line in gray represents the condition of perfect agreement between predicted and observed probabilities; Supplementary Figure S3: Calibration plot on PSM data corresponding to the death risk categories defined on ICSM data. The plot represents graphically the observed and predicted probabilities of OS for patients belonging to the low- and high-risk categories. The dashed line in gray represents the condition of perfect agreement between predicted and observed probabilities; Supplementary Table S1: Variables included in the multivariate predictive models and corresponding values: Lasso grouping indicates which variables have been grouped during the features selection process: metastases have been grouped so that all metastases sites could have been excluded or included together, but no subset of them could have been eliminated. For variables characterized by > 2 levels (IHC PDL1, ECOG PS, Charlson score), the corresponding dummy variables were also grouped to avoid the elimination of clinically meaningful variables levels; Supplementary Table S2: Variables binarized in each model tested. All starting models included all variables reported in Supplementary Table S1 (Age, Sex, Smoker habits, Histotype, Line of treatment, IHC PDL1, ECOG PS, LDH, NLR, Metastasis, Anemia, Thrombosis before therapy start, ACCI): different combinations of variables have been binarized in turn defining the 4 models reported. As an example: Model 1 included all variables coded according to their original values, model 2 included variables coded according to their original values except for IHC PDL1, which has been binarized as reported in Supplementary Table S1; Supplementary Table S3: Association between variables of interest and best response by cohort and in combined datasets; Variable = analyzed variable and corresponding values; PD = absolute and relative frequency (%) of patients by variable’s category among those with best response type PD; PR = absolute and relative frequency (%) of patients by variable’s category among those with best response type PR; SD = absolute and relative frequency (%) of patients by variable’s category among those with best response type SD. PD vs. PR/SD: results from logistic regression for PD vs. PR/SD outcome—OR (95% CI) = odds ratio from regression comparing the odds for PD between reference and baseline values and 95% confidence interval; *p* = *p*-value from logistic regression; in combined datasets analysis the center ID was used as a covariate to adjust for potential confounders# Including soft tissues and adrenal glands. Abbreviations: ADC, adenocarcinoma; ECOG PS, Eastern Cooperative Oncology Group Performance Status; IHC, immunohistochemical; CIRS, Cumulative Illness Rating Scale; NLR, neutrophil/lymphocyte ratio; SCC, squamocellular carcinoma; PD, progressive disease; PR, partial response; SD, stable disease; Supplementary Table S4: Progression-free survival analyses. Variable = analyzed variable; Value = values that each variable assumes; HR (95% CI) = Hazard Ratio and 95% CI from Cox regression; *p* = *p*-value from Cox regression. Results reported derive from univariate analyses except for combined cohorts analysis (§), where the center ID (ICSM/PSM) was included in the model to adjust for potential differences between cohorts. * *p* < 0.05 ^#^ Including: soft tissue and adrenal glands; Supplementary Table S5: Points generated by the PFS and OS nomograms to each variable’s value; Supplementary Table S6: Overall survival analyses. Variable = analyzed variable; Value = values that each variable assumes; HR (95% CI) = Hazard Ratio and 95% CI from Cox regression; *p* = *p*-value from Cox regression. Results reported derive from univariate analyses except for combined cohorts analysis (§), where the center ID (ICSM/PSM) was included in the model to adjust for potential differences between cohorts. * *p* < 0.05 ^#^ Including: soft tissue and adrenal gland.
