# Supplementary material for: Clinical and Biological Variables Influencing Outcome in Patients with Advanced Non-Small Cell Lung Cancer (NSCLC) Treated with Anti-PD-1/PD-L1 Antibodies: A Prospective Multicentre Study
Source: J Pers Med. 2022 Apr 24;12(5):679. doi: 10.3390/jpm12050679 (PMC9144987; doi:10.3390/jpm12050679)
Supplement: Supplementary file 1 [file jpm-12-00679-s001.zip › Supplementary Table S1.pdf]

**Lasso grouping**	**Variable**	**Original values**	**Binarized values**
Age	Age at treatment start (years)	< 65, ≥ 65	
Sex	Sex	Females, males	
Smoker habits	Smoker habits	Never, current/active	
Histotype	Histotype	ADC, SCC	
Line of treatment	Line of treatment	1, 2, 3, 4	
IHC PDL1	IHC PDL1 (%)	< 1%, 1 - 24%, 25 - 49%, ≥ 50%	< 25%, ≥ 25%
ECOG PS	ECOG PS	0, 1, 2-3	0 - 1, 2 - 3
LDH	LDH	< 325, ≥ 325	
NLR	NLR	< 5, ≥ 5	
Metastasis	Lung metastasis	No, Yes	
	Liver metastasis	No, Yes	
	Lymph nodes metastasis	No, Yes	
	Bone metastasis	No, Yes	
	Brain metastasis	No, Yes	
	Pleural metastasis	No, Yes	
	Other metastasis	No, Yes	
Anaemia	Anaemia	No, Yes	
Thrombosis before therapy start	Thrombosis before therapy start	No, Yes	
ACCI	ACCI (points)	< 9, ≥ 9	

Supplementary Table S1 – Variables included in the multivariate predictive models and corresponding values. Lasso grouping indicate which variables have been grouped during the features selection process: metastases have been grouped so that all metastases sites could have been excluded or included together but no subset of them could have been eliminated. For variables characterized by > 2 levels (IHC PDL1, ECOG PS, Charlson score) the corresponding dummy variables were also grouped to avoid the elimination of clinically meaningful variables levels.
