# Supplementary material for: Clinical and Biological Variables Influencing Outcome in Patients with Advanced Non-Small Cell Lung Cancer (NSCLC) Treated with Anti-PD-1/PD-L1 Antibodies: A Prospective Multicentre Study
Source: J Pers Med. 2022 Apr 24;12(5):679. doi: 10.3390/jpm12050679 (PMC9144987; doi:10.3390/jpm12050679)
Supplement: Supplementary file 1 [file jpm-12-00679-s001.zip › Supplementary Table S2.pdf]

Model	Binarized variables
1	-
2	IHC PDL1 (%)
3	ECOG PS
4	IHC PDL1 (%), ECOG PS

Supplementary Table S2 – Variables binarized in each model tested. All starting models included all variables reported in **Supplementary Table S1** (Age, Sex, Smoker habits, Histotype, Line of treatment, IHC PDL1, ECOG PS, LDH, NLR, Metastasis, Anaemia, Thrombosis before therapy start, ACCI): different combinations of variables have been binarized in turn defining the 4 models reported. As an example: model 1 included all variables coded according to their original values, model 2 includes variables coded according to their original values except for IHC PDL1 that has been binarized as reported in **Supplementary Table S1.**
