# Supplementary material for: Clinical and Biological Variables Influencing Outcome in Patients with Advanced Non-Small Cell Lung Cancer (NSCLC) Treated with Anti-PD-1/PD-L1 Antibodies: A Prospective Multicentre Study
Source: J Pers Med. 2022 Apr 24;12(5):679. doi: 10.3390/jpm12050679 (PMC9144987; doi:10.3390/jpm12050679)
Supplement: Supplementary file 1 [file jpm-12-00679-s001.zip › Supplementary Table S4.pdf]

Variable	Value	ICSM HR (95% CI)	ICSM p		PSM HR (95% CI)	PSM p		Combined § HR (95% CI)	Combined § p	
Line of treatment	1st	baseline			baseline			baseline		
	2nd	1.38 (0.50-3.85)	0.5380		0.33 (0.16-0.70)	0.0039	*	0.58 (0.35-0.97)	0.0377	*
	3rd	1.26 (0.41-3.90)	0.6827		0.14 (0.03-0.62)	0.0097	*	0.46 (0.23-0.90)	0.0244	*
	4th	1.06 (0.33-3.44)	0.9262		0.22 (0.05-1.01)	0.0514		0.43 (0.20-0.91)	0.0266	*
Sex	Female	baseline			baseline			baseline		
	Male	0.98 (0.59-1.64)	0.9412		12.34 (1.68-90.68)	0.0135	*	1.51 (0.94-2.44)	0.0897	
Smoker habits	Never	baseline			baseline			baseline		
	Former/active	1.65 (0.52-5.26)	0.3988		0.47 (0.14-1.61)	0.2310		1.08 (0.47-2.47)	0.8567	
Age at treatment start (years)	< 65	baseline			baseline			baseline		
	≥ 65	1 (0.61-1.65)	0.9872		0.79 (0.40-1.56)	0.5019		0.91 (0.61-1.36)	0.6593	
Histotype	ADC	baseline			baseline			baseline		
	SCC	1.37 (0.84-2.24)	0.2127		0.44 (0.15-1.25)	0.1244		1.05 (0.69-1.61)	0.8168	
IHC PDL1	< 1%	baseline			baseline			baseline		
	1-24%	0.44 (0.26-0.74)	0.0021	*	0.65 (0.14-2.94)	0.5759		0.49 (0.30-0.79)	0.0036	*
	25-49%	0.25 (0.11-0.56)	0.0008	*	0.51 (0.12-2.17)	0.3584		0.28 (0.14-0.55)	0.0002	*
	≥ 50%	0.18 (0.05-0.60)	0.0051	*	1.28 (0.38-4.30)	0.6850		0.51 (0.29-0.91)	0.0223	*
ECOG PS	0	baseline			baseline			baseline		
	1	0.60 (0.35-1.01)	0.0540		1.64 (0.81-3.32)	0.1722		0.83 (0.55-1.27)	0.3952	
	2-3	2.33 (1.19-4.55)	0.0138	*	4.80 (1.53-15.1)	0.0072	*	2.79 (1.56-4.99)	0.0006	*
Anaemia	no	baseline			baseline			baseline		
	yes	2.23 (1.39-3.58)	0.0009	*	3.86 (1.94-7.71)	0.0001	*	2.72 (1.84-4.04)	<0.0001	*
NLR		baseline			baseline			baseline		
	≥ 5	1.92 (1.19-3.09)	0.0077	*	2.93 (1.48-5.82)	0.0021	*	2.24 (1.51-3.33)	<0.0001	*
LDH		baseline			baseline			baseline		
	≥ 325	1.32 (0.81-2.14)	0.2663		1.11 (0.57-2.19)	0.7561		1.25 (0.84-1.85)	0.2651	
Lung metastases	no	baseline						baseline		
	yes	0.65 (0.36-1.16)	0.1420		-			0.63 (0.35-1.11)	0.1097	
Liver metastases	no	baseline			baseline			baseline		
	yes	0.90 (0.47-1.73)	0.7572		2.15 (0.51-9.12)	0.2999		1.02 (0.56-1.84)	0.9587	
Lymph nodes metastases	no	baseline			baseline			baseline		

	yes	0.94 (0.43-2.07)	0.8822		0.82 (0.34-1.97)	0.6499		0.89 (0.50-1.59)	0.6948	
Bone metastases	no	baseline			baseline			baseline		
	yes	0.90 (0.53-1.50)	0.6756		2.21 (0.91-5.36)	0.0781		1.09 (0.69-1.72)	0.7125	
Brain metastases	no	baseline			baseline			baseline		
	yes	1.40 (0.64-3.07)	0.4013		1.13 (0.49-2.60)	0.7673		1.23 (0.69-2.18)	0.4762	
Pleural metastases	no	baseline			baseline			baseline		
	yes	1.47 (0.77-2.79)	0.2442		6.06 (1.72-21.38)	0.0051	*	1.84 (1.03-3.29)	0.0382	*
Other metastases #	no	baseline			baseline			baseline		
	yes	1.07 (0.60-1.91)	0.8099		1.72 (0.84-3.52)	0.1372		1.28 (0.82-2)	0.2752	
Thrombosis before therapy	no	baseline			baseline			baseline		
	yes	0.92 (0.56-1.52)	0.7557		0.90 (0.12-6.63)	0.9197		0.93 (0.57-1.50)	0.7574	
ACCI	< 9	baseline			baseline			baseline		
	≥ 9	1.30 (0.81-2.07)	0.2784		0.88 (0.37-2.13)	0.7809		1.19 (0.78-1.82)	0.4087	
ICI toxicity	no	baseline			baseline			baseline		
	yes	0.92 (0.58-1.47)	0.7422		2.23 (0.92-5.41)	0.0762		1.12 (0.73-1.72)	0.6187	
Blood transfusions	no	baseline			baseline			baseline		
	yes	1.04 (0.45-2.4)	0.9331		1.18 (0.16-8.82)	0.8697		1.03 (0.48-2.24)	0.9354	
Oral or intravenous iron supplements	no	baseline			baseline			baseline		
	yes	0.67 (0.25-1.85)	0.4449		7.39 (1.63-33.45)	0.0094	*	1.03 (0.45-2.36)	0.9520	
Erytropoietin use	no	baseline			baseline			baseline		
	yes	2.28 (1.25-4.18)	0.0075	*	98.64 (5.92-1642.79)	0.0014	*	2.53 (1.41-4.54)	0.0018	*
Antibiotic use	no	baseline			baseline			baseline		
	yes	2.02 (1.05-3.9)	0.0354	*	1.95 (0.26-14.63)	0.5171		2.02 (1.08-3.77)	0.0272	*
Proton pump inhibitor use	no	baseline			baseline			baseline		
	yes	0.87 (0.53-1.42)	0.5803		1.74 (0.85-3.56)	0.1302		1.10 (0.74-1.65)	0.6334	
Antiplatelet/anticoagulant treatment	no	baseline			baseline			baseline		
	yes	1.64 (1.02-2.62)	0.0409	*	2.31 (1.18-4.54)	0.0151	*	1.79 (1.21-2.63)	0.0033	*
Steroid use	no	baseline			baseline			baseline		
	yes	2.36 (1.47-3.79)	0.0004	*	1.87 (0.57-6.18)	0.3037		2.30 (1.49-3.56)	0.0002	*

Supplementary Table S4 – Progression free survival analyses. Variable = analysed variable; Value = values that each variable assumes; HR (95% CI) = Hazard Ratio and 95% CI from Cox regression; p = p-value from Cox regression. Results reported derive from univariate analyses except for combined cohorts analysis (§) where the centre ID (ICSM/PSM) was included in the model to adjust for potential differences between cohorts.

* $p < 0.05$

Including: soft tissue and adrenal glands
