# Supplementary material for: Clinical and Biological Variables Influencing Outcome in Patients with Advanced Non-Small Cell Lung Cancer (NSCLC) Treated with Anti-PD-1/PD-L1 Antibodies: A Prospective Multicentre Study
Source: J Pers Med. 2022 Apr 24;12(5):679. doi: 10.3390/jpm12050679 (PMC9144987; doi:10.3390/jpm12050679)
Supplement: Supplementary file 1 [file jpm-12-00679-s001.zip › Supplementary Table S5.pdf]

	Points	
	**PFS**	**OS**
**IHC PDL1**		
< 1%	100	100
1-24%	74	70
25-49%	21	0
≥ 50%	0	23
**ECOG PS**		
0/1	0	0
2/3	61	61
**Anaemia**		
No	0	0
Yes	34	57
**NLR**		
< 5	0	0
≥ 5	25	23
**Risk categories based on total points**		
Low risk	< 104	< 87
High risk	≥ 104	≥ 87

Supplementary Table S5. Points generated by the PFS and OS nomograms to each variable's value.
