# Supplementary material for: Clinical and Biological Variables Influencing Outcome in Patients with Advanced Non-Small Cell Lung Cancer (NSCLC) Treated with Anti-PD-1/PD-L1 Antibodies: A Prospective Multicentre Study
Source: J Pers Med. 2022 Apr 24;12(5):679. doi: 10.3390/jpm12050679 (PMC9144987; doi:10.3390/jpm12050679)
Supplement: Supplementary file 1 [file jpm-12-00679-s001.zip › Supplementary Table S6.pdf]

		ICSM			PSM			Combined [§]		
**Variable**	**Value**	**HR (95% CI)**	**p**		**HR (95% CI)**	**p**		**HR (95% CI)**	**p**	
Line of treatment	1st	Baseline			Baseline			Baseline		
	2nd	0.77 (0.27-2.16)	0.6137		0.24 (0.10-0.58)	0.0016	*	0.36 (0.21-0.65)	0.0006	*
	3rd	0.91 (0.29-2.85)	0.8763		0.09 (0.01-0.67)	0.0190	*	0.36 (0.18-0.75)	0.0063	*
	4th	0.67 (0.20-2.19)	0.5034		0.25 (0.05-1.18)	0.0804		0.33 (0.15-0.71)	0.0048	*
Sex	Female	Baseline			Baseline			Baseline		
	Male	0.96 (0.56-1.65)	0.8784		8.72 (1.18-64.64)	0.0341	*	1.37 (0.83-2.26)	0.2248	
Smoker habits	Never	Baseline			Baseline			Baseline		
	Former/active	1.3 (0.41-4.15)	0.6614		0.27 (0.08-0.96)	0.0428	*	0.76 (0.33-1.76)	0.5271	
Age at treatment start (years)	< 65	Baseline			Baseline			Baseline		
	≥ 65	1.25 (0.74-2.11)	0.4029		0.69 (0.32-1.47)	0.3357		1.05 (0.69-1.61)	0.8164	
Histotype	ADC	Baseline			Baseline			Baseline		
	SCC	1.16 (0.70-1.92)	0.5766		0.41 (0.12-1.38)	0.1486		0.95 (0.60-1.49)	0.8180	
IHC PDL1	<1%	Baseline			Baseline			Baseline		
	1-24%	0.43 (0.25-0.75)	0.0031	*	52630538.98 (0-Inf)	0.9977		0.48 (0.28-0.81)	0.0065	*
	25-49%	0.24 (0.10-0.57)	0.0013	*	52424762.15 (0-Inf)	0.9977		0.30 (0.15-0.62)	0.0011	*
	≥ 50%	0.29 (0.09-0.95)	0.0408	*	98488450.37 (0-Inf)	0.9976		0.64 (0.33-1.22)	0.1761	
ECOG PS	0	Baseline			Baseline			Baseline		
	1	0.83 (0.49-1.42)	0.5025		1.34 (0.59-3.07)	0.4827		0.96 (0.61-1.51)	0.8649	
	2-3	2.66 (1.33-5.31)	0.0055	*	8.36 (2.44-28.61)	0.0007	*	3.48 (1.90-6.38)	<0.0001	
Anaemia	No	Baseline			Baseline			Baseline		
	Yes	2.66 (1.60-4.43)	0.0002	*	4.18 (1.87-9.37)	0.0005	*	3.02 (1.96-4.65)	<0.0001	
NLR	< 5	Baseline			Baseline			Baseline		
	≥ 5	2.14 (1.28-3.55)	0.0035	*	2.8 (1.28-6.16)	0.0103	*	2.30 (1.50-3.53)	0.0001	*
LDH	< 325	Baseline			Baseline			Baseline		
	≥ 325	1.81 (1.08-3.03)	0.0249	*	0.92 (0.42-1.99)	0.8237		1.47 (0.97-2.24)	0.0716	
Lung metastases	No	Baseline			Baseline			Baseline		
	Yes	0.78 (0.42-1.44)	0.4268		n.t.			0.76 (0.41-1.41)	0.3913	
Liver metastases	No	Baseline			Baseline			Baseline		
	Yes	0.86 (0.43-1.69)	0.6564		0.97 (0.13-7.28)	0.9792		0.86 (0.45-1.64)	0.6447	
Lymph nodes metastases	No	Baseline			Baseline			Baseline		

	Yes	0.87 (0.37-2.03)	0.7459		0.74 (0.28-1.95)	0.5366		0.80 (0.43-1.52)	0.5015	
Bone metastases	No	Baseline			Baseline			Baseline		
	Yes	1 (0.59-1.69)	0.9872		2.12 (0.8-5.62)	0.1325		1.15 (0.72-1.84)	0.5583	
Brain metastases	No	Baseline			Baseline			Baseline		
	Yes	1.82 (0.82-4.04)	0.1411		1.50 (0.63-3.58)	0.3571		1.63 (0.91-2.93)	0.1037	
Pleural metastases	No	Baseline			Baseline			Baseline		
	Yes	1.68 (0.87-3.23)	0.1230		6.98 (1.92-25.42)	0.0032	*	2.06 (1.15-3.72)	0.0157	*
Other metastases #	No	Baseline			Baseline			Baseline		
	Yes	1.33 (0.73-2.44)	0.3500		2.29 (1.04-5.03)	0.0397	*	1.62 (1.01-2.60)	0.0454	*
Thrombosis before therapy	No	Baseline			Baseline			Baseline		
	Yes	0.85 (0.51-1.42)	0.5343		0 (0-Inf)	0.9978		0.81 (0.49-1.35)	0.4244	
ACCI (points)	< 9	Baseline			Baseline			Baseline		
	≥ 9	1.37 (0.84-2.25)	0.2097		1.04 (0.36-3.01)	0.9463		1.32 (0.84-2.07)	0.2295	
ICI toxicity	No	Baseline			Baseline			Baseline		
	Yes	0.74 (0.46-1.21)	0.2334		2.04 (0.77-5.42)	0.1539		0.88 (0.55-1.39)	0.577	
Blood transfusions	No	Baseline			Baseline			Baseline		
	Yes	1.43 (0.62-3.32)	0.4032		1.07 (0.14-7.97)	0.9507		1.37 (0.63-2.98)	0.4206	
Oral or intravenous iron supplements	No	Baseline			Baseline			Baseline		
	Yes	0.51 (0.16-1.64)	0.2591		3.68 (0.85-15.86)	0.0803		0.79 (0.32-1.97)	0.6165	
Erythropoietin use	No	Baseline			Baseline			Baseline		
	Yes	2.67 (1.44-4.95)	0.0019	*	69.5 (4.35-1111.12)	0.0027	*	2.96 (1.63-5.38)	0.0004	*
Antibiotic use	No	Baseline			Baseline			Baseline		
	Yes	2.36 (1.21-4.58)	0.0116	*	5.66 (0.69-46.37)	0.1065		2.48 (1.32-4.69)	0.0050	*
Proton pump inhibitor use	No	Baseline			Baseline			Baseline		
	Yes	0.86 (0.52-1.43)	0.5625		1.48 (0.67-3.25)	0.33		1 (0.65-1.54)	0.9903	
Antiplatelet/anticoagulant treatment	No	Baseline			Baseline			Baseline		
	Yes	1.78 (1.08-2.92)	0.0231	*	2.30 (1.07-4.97)	0.0338	*	1.91 (1.25-2.9)	0.0025	*
Steroid use	No	Baseline			Baseline			Baseline		
	Yes	2.11 (1.29-3.45)	0.0029	*	1.98 (0.46-8.48)	0.357		2.1 (1.32-3.33)	0.0017	*

Supplementary Table S6 – Overall survival analyses. Variable = analysed variable; Value = values that each variable assumes; HR (95% CI) = Hazard Ratio and 95% CI from Cox regression; p = p-value from Cox regression. Results reported derive from univariate analyses except for combined cohorts analysis (§) where the centre ID (ICSM/PSM) was included in the model to adjust for potential differences between cohorts.

* $p < 0.05$

[#] Including: soft tissue and adrenal gland
