# Supplementary figures and images for: Clinical and Biological Variables Influencing Outcome in Patients with Advanced Non-Small Cell Lung Cancer (NSCLC) Treated with Anti-PD-1/PD-L1 Antibodies: A Prospective Multicentre Study
Source: J Pers Med. 2022 Apr 24;12(5):679. doi: 10.3390/jpm12050679 (PMC9144987; doi:10.3390/jpm12050679)

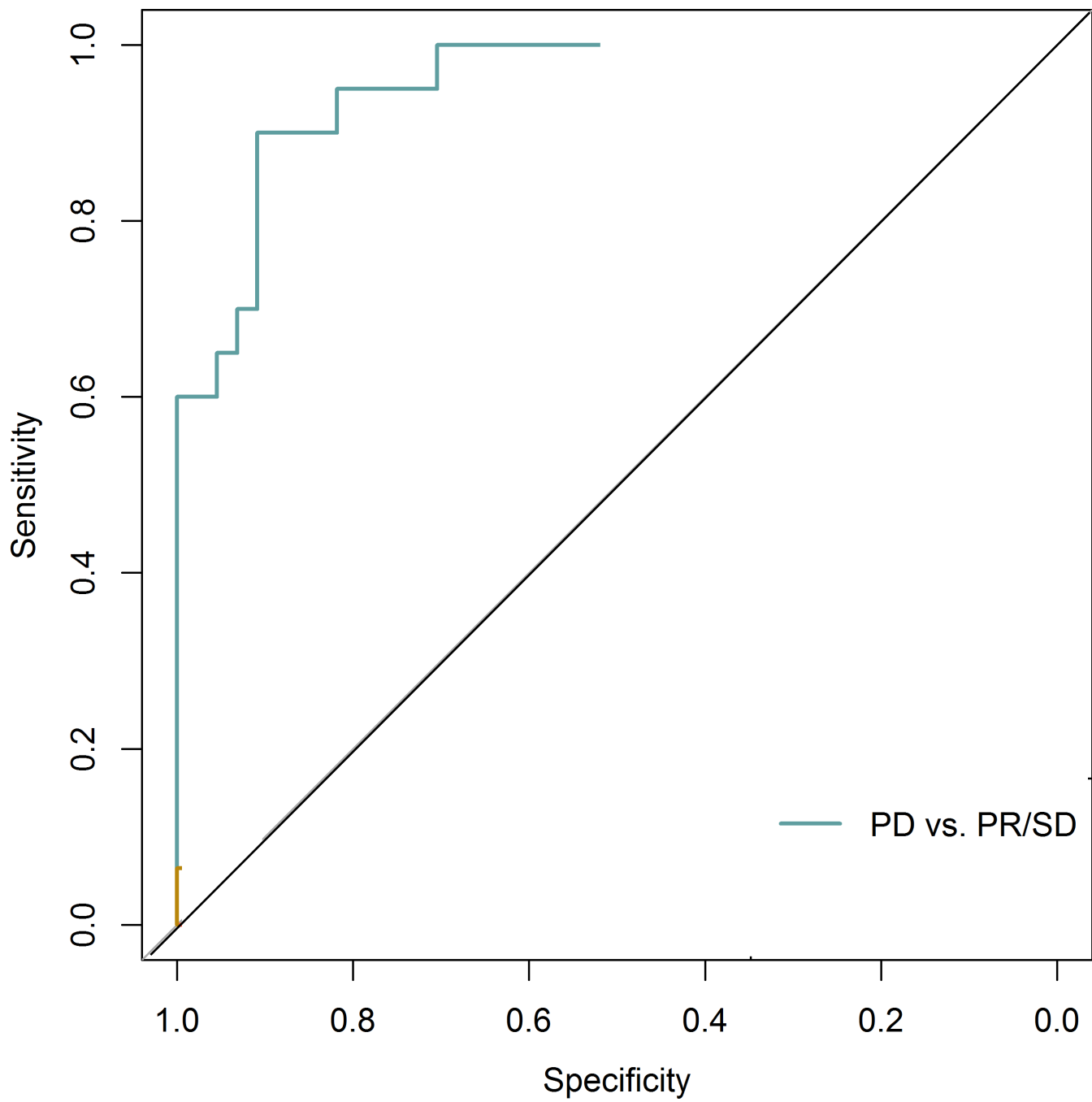

Supplement: Supplementary file 1 [file jpm-12-00679-s001.zip › Supplementary Figure S1.pdf]

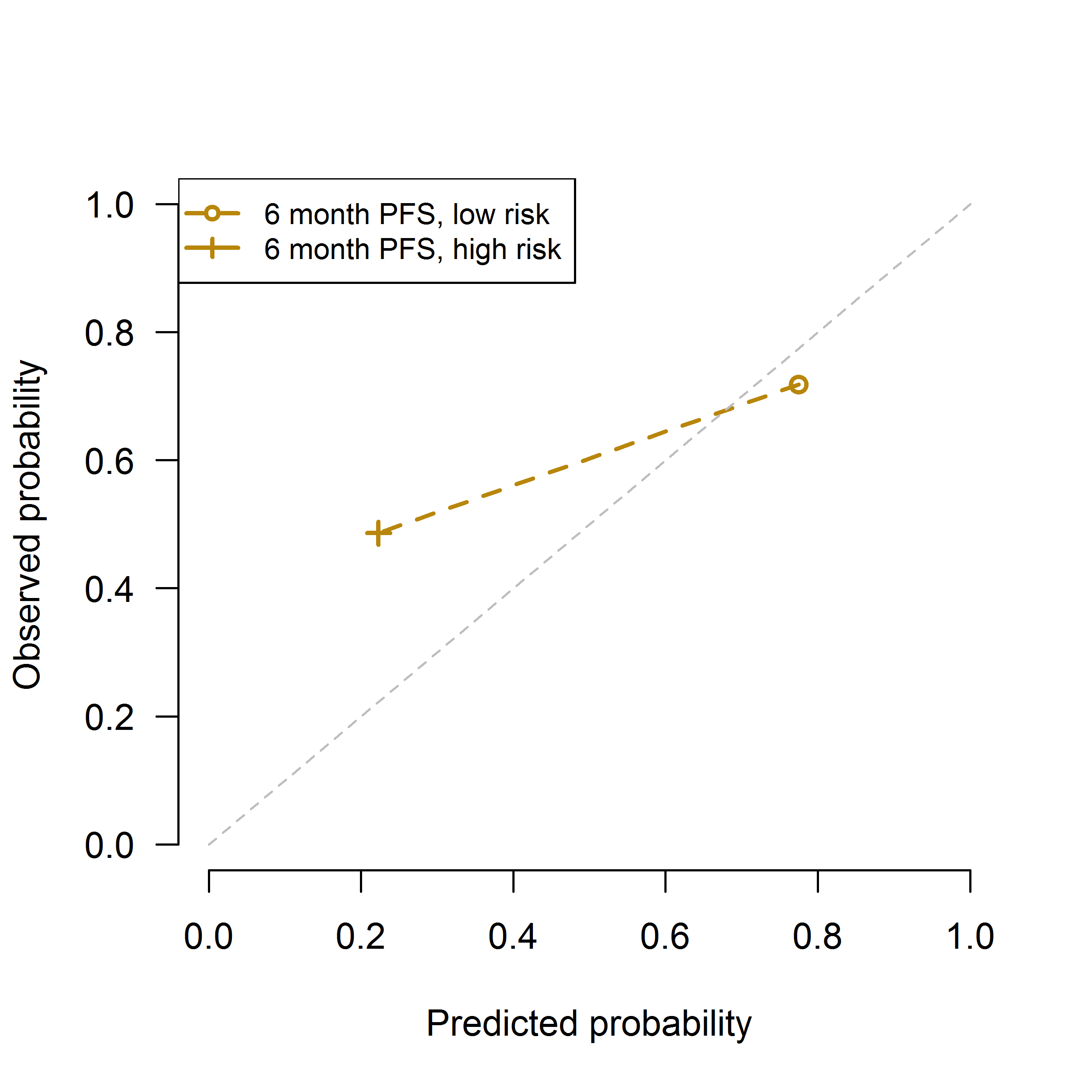

Supplement: Supplementary file 1 [file jpm-12-00679-s001.zip › Supplementary Figure S2.tiff]

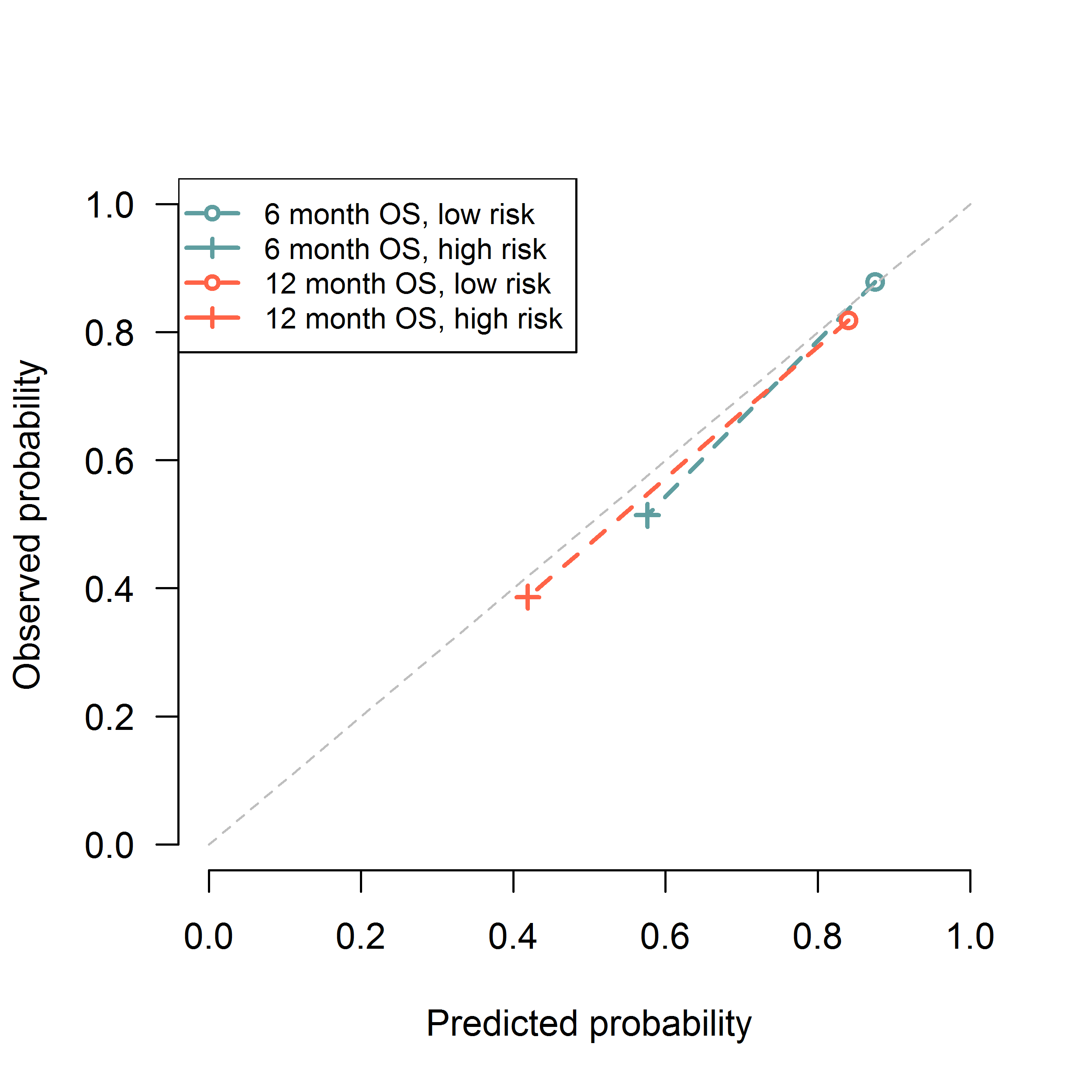

Supplement: Supplementary file 1 [file jpm-12-00679-s001.zip › Supplementary Figure S3.tiff]
